# Long-term efficacy and safety of subcutaneous tocilizumab in clinical trials of polyarticular or systemic juvenile idiopathic arthritis

**DOI:** 10.1093/rheumatology/keae180

**Published:** 2024-03-29

**Authors:** Hermine I Brunner, Nicolino Ruperto, Athimalaipet V Ramanan, Gerd Horneff, Kirsten Minden, Inmaculada Calvo Penades, Ekaterina Alexeeva, Gavin Cleary, Sara M Stern, Isabelle Kone-Paut, María del Rocío Maldonado Velázquez, C Egla Rabinovich, Agustin Remesal, Clovis Artur Silva, Irina Nikishina, Mauro Zucchetto, Laura Brockwell, Oliver Gordon, Sandra Nagel, Fabrizio De Benedetti, for the PRINTO and PRCSG Investigators, Rubén Cuttica, Maria Elena Rama, Jonathan Akikusa, Jeffrey Chaitow, Claudio Len, Clovis Artur Silva, Heinrike Schmeling, Rayfel Schneider, Isabelle Kone-Paut, Markus Hufnagel, Kirsten Minden, Gerd Horneff, Fabrizio de Benedetti, María del Rocío Maldonado Velázquez, Nadina Rubio, Ekaterina Alekseeva, Agustin Remesal, Alina Boteanu, Rosa Bou Torrent, Inmaculada Calvo Penades, Athimalaipet V Ramanan, Gavin Cleary, Hermine I Brunner, Ginger Janow, Jennifer Weiss, Daniel Lovell, Alan Martin, Kabita Nanda, Linda Wagner-Weiner, Sara Stern, Andrew Zeft, Jason Dare

**Affiliations:** Pediatric Rheumatology Collaborative Study Group (PRCSG), University of Cincinnati, Cincinnati Children’s Hospital Medical Center, Cincinnati, OH, USA; IRCCS Istituto Giannina Gaslini, UOC Servizio Sperimentazioni Cliniche Pediatriche/Gaslini Trial Centre, PRINTO, Genoa, Italy; Bristol Royal Hospital for Children and Translational Health Sciences, University of Bristol, Bristol, UK; Department of General Paediatrics, Asklepios Clinic Sankt Augustin, Sankt Augustin, Germany; Department of Pediatric and Adolescent Medicine, University Hospital of Cologne, Cologne, Germany; German Rheumatism Research Centre Berlin, Berlin, Germany; Department of Pediatric Respiratory Medicine, Immunology and Critical Care Medicine, Charité Universitätsmedizin, Berlin, Germany; Paediatric Rheumatology Unit, Hospital Universitario y Politécnico La Fe, València, Spain; National Medical Research Center of Children’s Health, Moscow, Russia; First Moscow State Medical University, Moscow, Russia; Paediatric Rheumatology, Alder Hey Children’s NHS Foundation Trust, Liverpool, UK; Division of Pediatric Rheumatology, Department of Pediatrics, University of Utah School of Medicine, Salt Lake City, UT, USA; European Reference Network for Immunodeficiency, Autoinflammatory, Autoimmune, and Paediatric Rheumatic Diseases (ERN-RITA) Member, Pediatric Rheumatology and, Bicêtre Hospital AP-HP, Centre de Référence des Maladies Autoinflammatoires et des Amyloses (CéRéMAIA), Paris, France; Hospital Infantil de México Federico Gómez, Distrito Federal, México; Department of Pediatrics, Duke University Medical Center, Durham, NC, USA; Pediatric Rheumatology Unit, University Hospital La Paz, Madrid, Spain; Pediatric Rheumatology Unit, Hospital das Clínicas HCFMUSP, Faculdade de Medicina, Universidade de São Paulo, São Paulo, Brazil; Pediatric Department, V.A. Nasonova Research Institute of Rheumatology, Moscow, Russian Federation; Parexel International, Milan, Italy; Roche Products Ltd, Welwyn Garden City, UK; Roche Products Ltd, Welwyn Garden City, UK; Roche Pharmaceutical Research and Early Development, Roche Innovation Center, Basel, Switzerland; Division of Rheumatology, Ospedale Pediatrico Bambino Gesù, IRCCS, Rome, Italy

**Keywords:** autoinflammatory conditions, juvenile idiopathic arthritis, paediatric/juvenile rheumatology, biologic therapies, pharmacology, interleukin-6, tocilizumab, clinical trial, long-term extension, pharmacovigilance

## Abstract

**Objective:**

To investigate the safety and efficacy of subcutaneous tocilizumab (SC-TCZ) treatment in a long-term extension (LTE) of clinical trials in polyarticular or systemic juvenile idiopathic arthritis (pJIA or sJIA).

**Methods:**

Patients with pJIA or sJIA from two open-label, 52-week phase 1b core trials of SC-TCZ who had adequate response per investigator assessment entered the LTE and continued SC-TCZ treatment according to body weight–based dosing regimens until commercial availability or up to 5 years. Pharmacokinetics, pharmacodynamics, and efficacy were assessed for up to 3 years, and safety for up to 5 years in the LTE.

**Results:**

Forty-four patients with pJIA and 38 patients with sJIA entered the LTE. Tocilizumab trough concentrations were maintained within the range expected to provide clinical benefit (mean values: pJIA, ∼10 μg/ml; sJIA, ∼75 μg/ml over 3 years). Pharmacodynamic parameters (interleukin-6, soluble interleukin-6 receptor, erythrocyte sedimentation rate, C-reactive protein) were maintained throughout the LTE at levels achieved in the core trials. Inactive disease per American College of Rheumatology provisional criteria was reported for 90% (17/19) and 53% (8/15) of patients with pJIA and 91% (10/11) and 92% (12/13) of patients with sJIA in the <30 and ≥30 kg body weight groups, respectively. Serious adverse events in the LTE were reported in six patients with pJIA (13.6%; five serious infections) and five patients with sJIA (13.2%; one serious infection).

**Conclusion:**

Patients with pJIA or sJIA experienced long-term disease control with SC-TCZ treatment. Long-term safety was consistent with the known tocilizumab safety profile.

**Clinical trial registration:**

clinicaltrials.gov, NCT02165345

Rheumatology key messages

- Patients with polyarticular/systemic juvenile idiopathic arthritis experienced long-term disease control with subcutaneous tocilizumab.
- Pharmacokinetics and pharmacodynamics of tocilizumab were within ranges indicative of clinical efficacy.
- Long-term safety was consistent with the tocilizumab safety profile, with no new safety signals.

## Introduction

Juvenile idiopathic arthritis (JIA) is a heterogeneous group of diseases characterized by arthritis with onset before 16 years of age that persists for longer than 6 weeks [1]. The recommended course of treatment depends on the JIA category. Early initiation of methotrexate, with the addition of a biologic disease-modifying antirheumatic drug including an interleukin-6 inhibitor for additional disease control in patients with moderate or high disease activity, is suggested in the JIA ACR guidelines [2, 3].

Interleukin-6 levels are elevated in the serum and synovial fluid in polyarticular JIA (pJIA) [4], and especially high in systemic JIA (sJIA) [5, 6]. Tocilizumab, a humanized monoclonal anti–interleukin-6 receptor alpha antibody that blocks the activity of interleukin-6, is indicated as an intravenous or subcutaneous formulation for the treatment of adult and pediatric rheumatic and inflammatory diseases, including pJIA and sJIA [1].

Subcutaneous dosing of tocilizumab for the treatment of children with pJIA and sJIA was previously determined by bridging pharmacokinetic, efficacy, and safety data from two open-label phase 1b core trials of subcutaneous tocilizumab (SC-TCZ; clinicaltrials.gov, NCT01904292 and NCT01904279) to data from phase 3 trials of intravenous tocilizumab [7]. This bridging analysis circumvented potential ethical challenges associated with conducting a placebo-controlled trial in children [8] and provided an option for the administration of tocilizumab that can be carried out by a parent or caregiver and does not require hospital visits, which can be worrisome for younger children and caregivers and may result in absence from school or work [9–11]. The SC-TCZ dosing regimens determined by the bridging analysis were approved by the US Food and Drug Administration, the European Medicines Agency, and other health authorities for the treatment of sJIA and pJIA.

We report the long-term efficacy and safety of SC-TCZ in patients with pJIA and sJIA from the long-term extension (LTE) of the phase 1b SC-TCZ core trials.

## Methods

### Patients

Patients with pJIA and sJIA classified according to the International League of Associations for Rheumatology criteria [12] from the two 52-week phase 1b trials [7] were followed for up to 3 additional years in the LTE for pharmacokinetic, pharmacodynamic, efficacy, and safety assessments. The LTE was extended for up to 5 years allowing patients who completed the study before tocilizumab became commercially available to have continued access for a maximum of 5 years or until commercial availability, whichever was earlier. Safety assessment was extended for up to 5 years, accordingly.

In the core pJIA trial, patients received open-label SC-TCZ 162 mg with a dosing interval based on body weight groups (every 3 weeks [Q3W] if patients weighed <30 kg or every 2 weeks [Q2W] if they weighed 30 kg or more). In the core sJIA trial, patients received open-label SC-TCZ 162 mg either every 10 days (Q10D) or Q2W if they weighed <30 kg (some patients modified their dosing regimen from Q10D to Q2W following a planned interim analysis) or weekly (QW) if they weighed 30 kg or more [7]. All patients who completed 52 weeks of treatment in the core trials and had adequate disease control with subcutaneous tocilizumab treatment (comparable to the use of intravenous tocilizumab if received before enrolment in the core trial) according to the clinical judgement of the investigator were eligible to enter the LTE and continue open-label SC-TCZ 162 mg according to their body weight dosing group schedule.

All patients or parents/guardians provided written informed consent/assent to participate in the trials, which were conducted in accordance with the Declaration of Helsinki and Good Clinical Practice guidelines. The protocols were approved by the institutional review boards or independent ethics committees at each study site (ethics approval number 2014–3379 for the Cincinnati Children’s Hospital Medical Center ethics committee at the primary site for Dr Brunner).

### Assessments

Pharmacokinetics were analyzed in the pharmacokinetic-evaluable population (patients who received ≥1 dose of SC-TCZ and had ≥1 post-dose pharmacokinetic assessment in the LTE). Pharmacokinetic parameters were exploratory endpoints assessed for up to 3 years in the LTE as steady-state serum tocilizumab concentration (C_trough_) measured at regular, prespecified intervals (baseline, every 24 weeks until week 144, first withdrawal visit) using validated enzyme-linked immunosorbent assay at a central laboratory (Quest Pharmaceutical Services, The Netherlands) for the different dosing regimens.

Pharmacodynamics were analyzed in the intention-to-treat population (patients who received ≥1 dose of SC-TCZ in the LTE) as exploratory endpoints for up to 3 years in the LTE as the change in serum interleukin-6, soluble interleukin-6 receptor (sIL-6R), C-reactive protein (CRP), and erythrocyte sedimentation rate (ESR).

Immunogenicity was assessed as the development of anti-tocilizumab antibodies assayed at prespecified intervals using a tiered testing strategy [13].

Efficacy was analyzed in the intention-to-treat population for up to 3 years in the LTE. Disease activity was measured as the change in Juvenile Arthritis Disease Activity Score on 71 joints (JADAS-71, range 0–101), and cutoff scores used for pJIA were: high disease activity, >10.5; moderate disease activity, 3.9–10.5; low disease activity, ≤3.8; inactive disease, ≤1 [14]. The proportion of patients with inactive disease was determined according to ACR provisional criteria [15, 16], with clinical remission on medication defined as inactive disease for ≥6 months continuously while receiving treatment. Health-related quality-of-life measures were physical function, assessed by the cross-culturally adapted and validated version of the Childhood Health Assessment Questionnaire-Disability Index (CHAQ-DI) [17], patient/parent assessment of pain, and patient/parent global evaluation of well-being. For analysis of JADAS-71, inactive disease, and clinical remission, the last observation carried forward of the last post-baseline value was applied to core set components.

Safety was assessed in the safety population (patients who received ≥1 dose of SC-TCZ and had ≥1 post-dose safety assessment in the LTE) for up to 5 years in the LTE as the incidence and rates of adverse events (AEs), serious AEs (SAEs), and adverse events of special interest (AESIs), with severity graded according to the Common Terminology Criteria for Adverse Events (v4.0). AESIs included serious infections, opportunistic infections, hypersensitivity reactions (defined as any AE occurring within 24 h of tocilizumab treatment and not considered ‘unrelated to’ study medication by the investigator, regardless of whether it was clinically consistent with hypersensitivity reaction), anaphylactic reactions, serious hepatic adverse events, gastrointestinal perforations, demyelinating disorders, myocardial infarction, stroke, serious bleeding events, malignancies, and suspected transmission of an infectious agent by the study drug. *Ad hoc* analysis was performed for potential cases of drug reaction with eosinophilia and systemic symptoms (DRESS), which is a potentially life-threatening syndrome usually characterized by delayed onset (2–8 weeks after receiving the drug), and clinical manifestations, including fever, rash, haematological abnormalities, and lymphadenopathy. Potential DRESS events were retrieved using the standardized Medical Dictionary for Regulatory Activities query narrow or wide for DRESS in an algorithmic search. Identified potential cases were then reviewed further to assess whether the course of events suggested the occurrence of DRESS according to the European Registry of Severe Cutaneous Adverse Reactions criteria [18], which propose hospitalization and reaction suspected to be drug-related as prerequisites for further application of criteria.

## Results

### Patients

**Figure 1.**
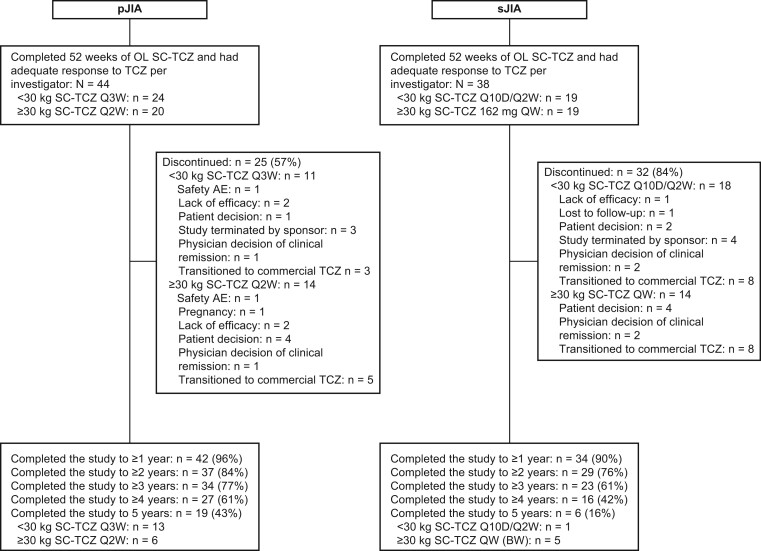
Patient disposition in the LTE study. Per protocol, patient participation in the study continued until tocilizumab became commercially available in a country or region or for a maximum of 5 years. Some patients with sJIA weighing <30 kg changed their dosing regimen from Q10D to Q2W following planned interim analysis. AE: adverse event; BW: body weight; LTE: long-term extension; OL: open-label; pJIA: polyarticular juvenile idiopathic arthritis; Q10D: every 10 days; Q2W: every 2 weeks; Q3W: every 3 weeks; QW: weekly; SC: subcutaneous; sJIA: systemic juvenile idiopathic arthritis; TCZ: tocilizumab

The LTE included 44 patients with pJIA (24 weighing <30kg received SC-TCZ Q3W; 20 weighing ≥30 kg received SC-TCZ Q2W) and 38 patients with sJIA (19 weighing <30 kg received SC-TCZ Q10D/Q2W; 19 weighing ≥30 kg received SC-TCZ QW) (Fig. 1). Among patients with pJIA, 19 (43%) completed the LTE study to 5 years (13 weighing <30 kg received TCZ Q3W; six weighing ≥30 kg received TCZ Q2W) and, among patients with sJIA, six (16%) completed the LTE study to 5 years (one weighing <30 kg received SC-TCZ Q10D/Q2W; five weighing ≥30 kg received SC-TCZ QW). The main reason for LTE discontinuation was the use of commercial tocilizumab as stipulated in the protocols (eight pJIA; 16 sJIA). Other reasons were physician decision due to disease remission (two pJIA; four sJIA), safety reasons/adverse events (one grade 4 neutropenia; one grade 2 JIA flare), pregnancy (one pJIA), and loss to follow-up (one pJIA). Five patients discontinued because of lack of efficacy (four pJIA; one sJIA), 11 discontinued due to patient/parent decision (five pJIA; six sJIA), and the remainder transitioned to post-trial access programs (three pJIA; four sJIA). There was no imputation of missing data for patients who discontinued early.

Demographics and disease characteristics upon entry to the LTE (LTE baseline) were similar between dosing groups, except for differences expected in body weight–based dosing such as age and weight (Table 1), and were generally comparable to baseline characteristics at entry into the core studies [7]. At LTE baseline, oral glucocorticoid use was reported in five patients with pJIA (11%) and six patients with sJIA (16%) at a mean (SD) daily dose of 0.11 (0.04) mg/kg/day and 0.31 (0.51) mg/kg/day, respectively; methotrexate use was reported in 27 patients with pJIA (61.4%) and 13 patients with sJIA (32.2%). Disease activity was low at LTE baseline (median JADAS-71: 1.1 for pJIA and 0.3 for sJIA), as expected because patients had received 52 weeks of tocilizumab treatment in the core studies and had to have an adequate response according to the investigator to enter the LTE.

**Table 1.** Demographics and disease characteristics at entry to the LTE study (LTE baseline)

	pJIA (*N *=* *44)		sJIA (*N *=* *38)	
	<30 kg SC-TCZ Q3W *n *=* *24	≥30 kg SC-TCZ Q2W *n *=* *20	<30 kg SC-TCZ Q10D/Q2W *n *=* *19	≥30 kg SC-TCZ QW *n *=* *19
Age, mean (sd), years	6.8 (2.1)	14.7 (2.8)	4.9 (2.3)	13.9 (3.5)
Female, *n* (%)	18 (75)	14 (70)	10 (53)	11 (58)
Weight, mean (sd), kg	24.1 (5.9)	60.4 (15.0)	19.5 (5.2)	51.1 (13.9)
Race, *n* (%)				
White	20 (83.3)	19 (95.0)	16 (84.2)	16 (84.2)
Other/unknown	4 (16.7)	1 (5.0)	3 (15.8)	3 (15.8)
Ethnicity, *n* (%)				
Hispanic or Latino	6 (25.0)	3 (15.0)	3 (15.8)	0
Not Hispanic or Latino	14 (58.3)	17 (85.0)	15 (78.9)	15 (78.9)
Not reported or unknown	4 (16.7)	0	1 (5.3)	4 (21.1)
Glucocorticoid use, *n* (%)	1 (4.2)	4 (20.0)	4 (21.1)	2 (10.5)
Daily dose of prednisone, mg/kg				
Mean (sd)	0.13 (NA)	0.10 (0.05)	0.49 (0.63)	0.03 (0.03)
Median (range)	0.13 (NA)	0.09 (0.05–0.17)	0.22 (0.03–1.21)	0.03 (0.02–0.05)
Methotrexate use, *n* (%)	14 (58.3)	13 (65.0)	6 (31.6)	7 (36.8)
Physician global assessment of disease activity VAS (0–100 mm)				
Mean (sd)	3.0 (6.6)	9.5 (16.4)	4.0 (9.2)	2.1 (4.9)
Median (range)	0.0 (0–28)	4.0 (0–73)	0.0 (0–39)	0.0 (0–21)
Patient/parent global assessment VAS (0–100 mm)				
Mean (sd)	7.8 (13.7)	15.2 (15.0)	6.6 (12.2)	6.4 (10.0)
Median (range)	1.5 (0–56)	14.0 (0–61)	1.0 (0–45)	1.0 (0–34)
Patient/parent pain VAS (0–100 mm)				
Mean (sd)	8.1 (15.2)	15.7 (16.8)	4.5 (9.5)	5.2 (8.6)
Median (range)	1.0 (0–72)	10.5 (0–57)	0.0 (0–33)	2.0 (0–36)
Pain VAS ≤35 mm, *n* (%)	23 (95.8)	17 (85.0)	19 (100)	18 (94.7)
CHAQ-DI (0–3)				
Mean (sd)	0.2 (0.4)	0.5 (0.8)	0.1 (0.2)	0.2 (0.5)
Median (range)	0.0 (0.0–1.8)	0.1 (0.0–2.8)	0.0 (0.0–0.5)	0.0 (0.0–2.3)
CHAQ-DI = 0, *n* (%)	16 (66.7)	6 (30.0)	13 (68.4)	15 (78.9)
ESR, mm/h				
Mean (sd)	3.6 (3.3)	4.6 (3.2)	5.9 (13.4)	2.5 (1.1)
Median (range)	2.0 (1.0–15.0)	4.0 (1.0–12.0)	2.0 (0.0–61.0)	2.0 (1.0–5.0)
Joints with active arthritis (0–71)				
Mean (sd)	0.5 (2.0)	2.7 (5.1)	0.3 (0.8)	0.5 (1.8)
Median (range)	0.0 (0–10)	0.0 (0–22)	0.0 (0–3)	0.0 (0–8)
JADAS-71, median (range)	0.4 (0.0–18.4)	2.1 (0.0–27.6)	0.4 (0.0–14.2)	0.3 (0.0–11.2)

JADAS-71: Juvenile Arthritis Disease Activity Score on 71 joints; LTE: long-term extension; NA: not applicable; pJIA: polyarticular juvenile idiopathic arthritis; Q10D: every 10 days; Q2W: every 2 weeks; Q3W: every 3 weeks; QW: weekly; SC: subcutaneous; sJIA: systemic juvenile idiopathic arthritis; TCZ: tocilizumab; VAS: visual analogue scale.

Background oral glucocorticoid use at any time during the study was reported in 39% (17/44) of patients with pJIA and 29% (11/38) of patients with sJIA, and methotrexate use was reported in 61% (27/44) and 34% (13/38), respectively.

### Pharmacokinetics and pharmacodynamics

**Figure 2.**
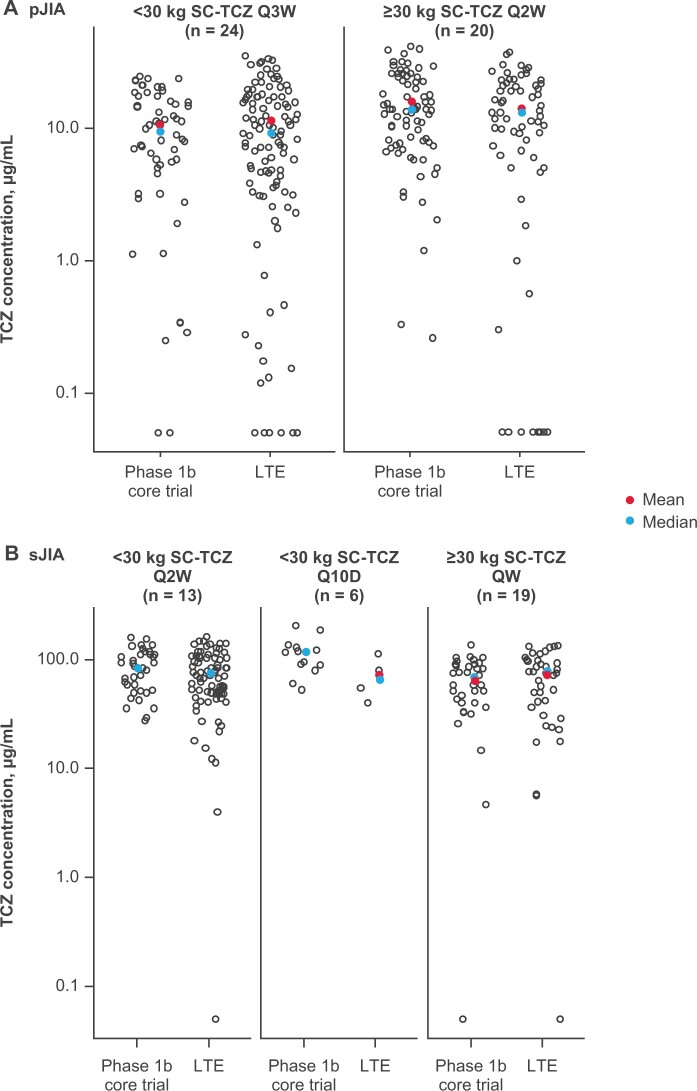
Tocilizumab trough concentrations among (A) patients with pJIA and (B) patients with sJIA (pharmacokinetic population). Data are shown as individual TCZ C_trough_ results (open circles) collected in the core trials (for patients who transitioned to the LTE) or in the LTE. C_trough_: trough concentration; LTE: long-term extension; pJIA: polyarticular juvenile idiopathic arthritis; Q10D: every 10 days; Q2W: every 2 weeks; Q3W: every 3 weeks; QW: weekly; sJIA: systemic juvenile idiopathic arthritis; TCZ: tocilizumab

Pharmacokinetic analysis was performed using a database of 763 samples collected from all 44 patients with pJIA and all 38 patients with sJIA in the LTE. Tocilizumab trough concentrations (C_trough_) during the LTE were maintained within the range expected to provide clinical benefit, with mean values of ∼10 μg/ml in pJIA and ∼75 μg/ml in sJIA. There was a similar spread of concentrations across the body weight dosing groups in each JIA subtype and between dosing groups in the LTE and the corresponding group in the core trials (Fig. 2).

**Figure 3.**
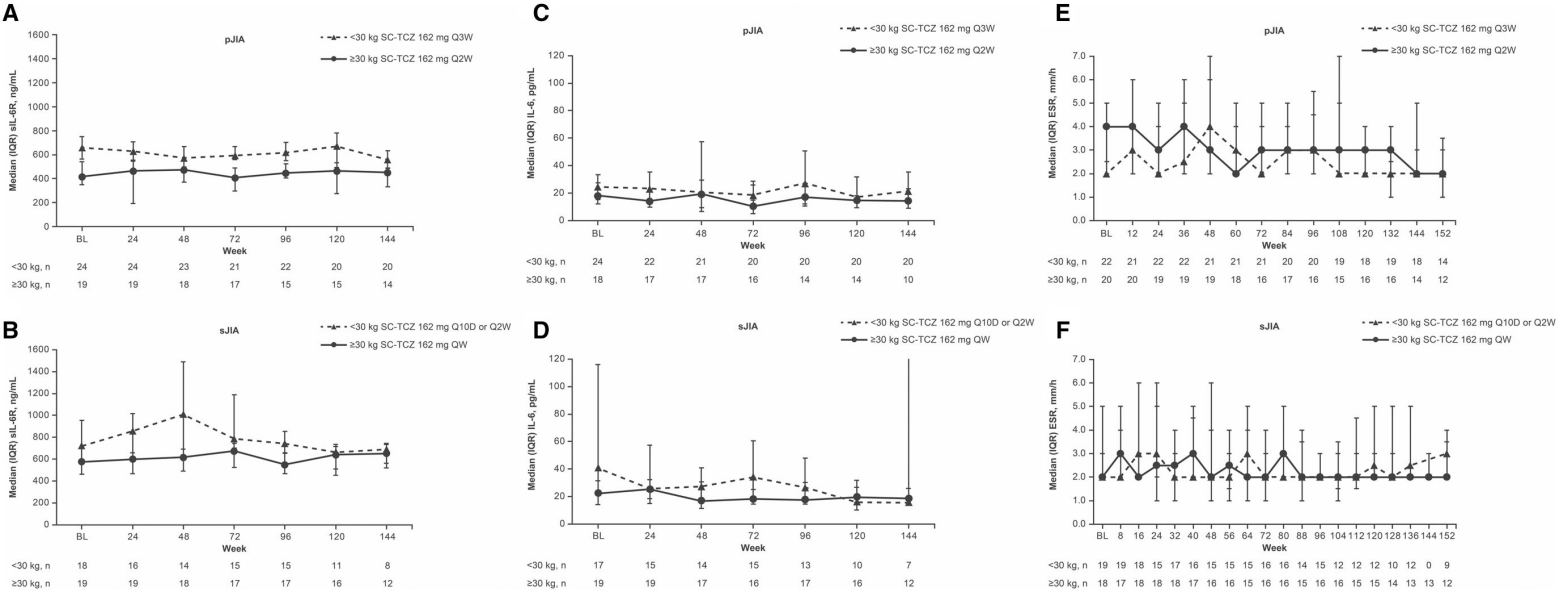
sIL-6R (A, B), IL-6 (C, D), and ESR (E, F) in the LTE (intention-to-treat population). BL is the last observation before initiation of study treatment in the LTE. Patients without BL assessment were excluded. The 75th percentile for interleukin-6 in patients with sJIA at week 144 is not shown because it is outside the limits of the graph (value = 322.0 pg/ml). BL: baseline; ESR: erythrocyte sedimentation rate; IQR: interquartile range; LTE: long-term extension; pJIA: polyarticular juvenile idiopathic arthritis; Q10D: every 10 days; Q2W: every 2 weeks; Q3W: every 3 weeks; QW: weekly, SC: subcutaneous; sIL-6R: soluble interleukin-6 receptor; sJIA: systemic juvenile idiopathic arthritis; TCZ: tocilizumab

sIL-6R and interleukin-6 levels were steady over time in the pJIA and sJIA groups (Fig. 3A, B). ESR was low at baseline in both JIA groups and was maintained to the end of the LTE assessment period (Fig. 3C). CRP levels were within the normal range for JIA (<10 mg/l) [14] at LTE baseline and remained low and within the normal range (median CRP <0.2 mg/l at all time points), with only minor fluctuations in all body weight dosing groups.

### Immunogenicity

Post-baseline evaluable samples for measurement of anti-tocilizumab antibodies were available for 41 patients with pJIA and 37 patients with sJIA. Two patients with pJIA (both in the ≥30 kg SC-TCZ Q2W group) developed anti-tocilizumab antibodies with neutralizing potential. One, who was concomitantly receiving methotrexate, tested positive for neutralizing anti-tocilizumab antibodies at weeks 48 and 72, but there was no impact on pharmacokinetics; they did not experience any hypersensitivity reactions or injection site reactions, completed the study, and there was no discernable impact on JADAS-71. The other patient, who was not receiving methotrexate, had one positive test result for neutralizing anti-tocilizumab antibodies at week 48; the impact on tocilizumab exposure could not be determined for this patient because the week 24 pharmacokinetic analysis sample had to be excluded due to compliance issues, leaving only one interpretable data point for week 48. However, a potential impact on exposure could not be excluded. This patient experienced several grade 1 injection site reactions that did not lead to treatment interruption, but there were no hypersensitivity reactions. Treatment was discontinued at week 64 because of lack of efficacy. No patients with sJIA developed anti-tocilizumab antibodies.

### Efficacy

**Figure 4.**
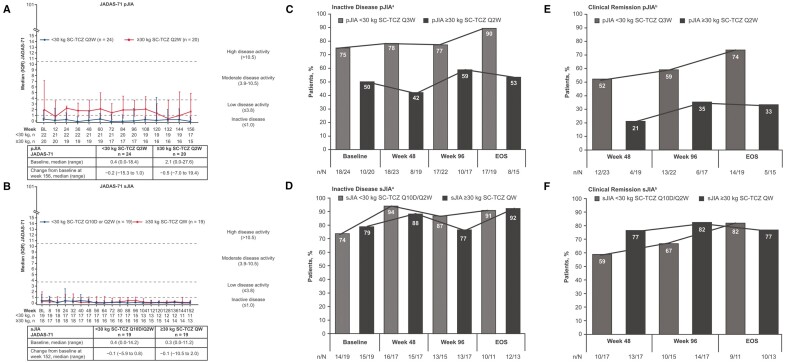
JADAS-71 (A, B), inactive disease (C, D), and clinical remission (E, F) (LTE intention-to-treat population). Horizontal lines connecting bars indicate the trend over time. Last observation carried forward was applied to JADAS-71 components that were missing. ^a^Inactive disease was defined according to Wallace criteria as no presence of active joints; absence of uveitis; no fever, rash, serositis, splenomegaly, hepatomegaly, or generalized lymphadenopathy attributable to pJIA or sJIA; erythrocyte sedimentation rate <20 mm/h; physician global visual analogue scale score ≤10 mm; and duration of morning stiffness ≤15 minutes. ^b^Clinical remission was defined as inactive disease for a minimum of 6 continuous months irrespective of disease-modifying antirheumatic drug, nonsteroidal anti-inflammatory drug, or glucocorticoid use. EOS: end of study efficacy assessment (week 156 for pJIA, week 152 for sJIA); JADAS-71: Juvenile Arthritis Disease Activity Score on 71 joints (maximum value = 71); *n*/*N*: number of patients meeting criteria/total number of patients with assessment; LTE: long-term extension; pJIA: polyarticular juvenile idiopathic arthritis; Q10D: every 10 days; Q2W: every 2 weeks; Q3W: every 3 weeks; QW: weekly; sJIA: systemic juvenile idiopathic arthritis; TCZ: tocilizumab

The median JADAS-71 remained stable over the course of the LTE (Fig. 4A, B). The median (range) improvement from baseline to week 156 in pJIA was −0.2 (−15.3–1.0) in the <30 kg SC-TCZ Q3W group and −0.5 (−7.0–19.4) in the ≥30 kg SC-TCZ Q2W group and the improvement from baseline to week 152 in sJIA was −0.1 (−5.9–0.8) in the <30 kg SC-TCZ Q10D/Q2W group and −0.1 (−10.5–2.0) in the ≥30 kg SC-TCZ QW group.

The proportion of patients with inactive disease (ACR provisional criteria) and clinical remission on medication (inactive disease for ≥6 months continuously) [16] remained stable in the LTE (Fig. 4C, D). Among patients with pJIA, inactive disease was achieved by 75% (18/24) in the <30 kg SC-TCZ Q3W group and 50% (10/20) in the ≥30 kg SC-TCZ Q2W group at LTE baseline and 90% (17/19) and 53% (8/15), respectively, at the end of the study (week 156). Clinical remission on medication was achieved by 74% (14/19) of patients with pJIA in the <30 kg SC-TCZ Q3W group and 33% (5/15) in the ≥30 kg SC-TCZ Q2W group at week 156. Among patients with sJIA, inactive disease was achieved by 74% (14/19) in the <30 kg SC-TCZ Q3W group and 79% (15/19) in the ≥30 kg SC-TCZ QW group at the LTE baseline and by 91% (10/11) and 92% (12/13), respectively, at the end of the study at week 152. Clinical remission on medication was achieved by 82% (9/11) of patients with sJIA in the <30 kg SC-TCZ Q3W group and 77% (10/13) in the ≥30 kg SC-TCZ QW group at week 152.

CHAQ-DI scores were low at LTE baseline and remained stable in the LTE study in both body weight dosing groups in pJIA and sJIA (Supplementary Fig. S1A and B, available at *Rheumatology* online). Absence of disability (CHAQ-DI = 0) among patients who had assessments at the end of the study was reported by 72% (18/25) of those with pJIA (<30 kg SC-TCZ Q3W, 9/13 [69%]; ≥30 kg SC-TCZ Q2W, 9/12 [75%]) at week 156 and by 74% (14/19) of those with sJIA (<30 kg SC-TCZ Q3W, 3/6 [50%]; ≥30 kg SC-TCZ QW, 11/13 [85%]) at week 152. The proportion of patients with assessments at the end of the study who had no more than minimal pain (pain VAS ≤35 mm) was 96% (24/25) for pJIA (<30 kg SC-TCZ Q3W, 13/13 [100%]; ≥30 kg SC-TCZ Q2W, 11/12 [92%]) at week 156 and 95% (18/19) for sJIA (<30 kg SC-TCZ Q3W, 5/6 [83%]; ≥30 kg SC-TCZ QW, 13/13 [100%]) at week 152. Patient/parent global assessment of disease activity remained consistently low during the LTE (Supplementary Fig. S2A and B, available at *Rheumatology* online).

### Safety

Among patients who entered the LTE, the median duration of treatment with SC-TCZ at the end of the LTE was 4.6 years (range, 0.3–5.1 years) for pJIA (5.0 [0.6–5.1] years in the <30 kg group and 4.1 [0.3–5.1] years in the ≥30 kg group) and 3.4 years (range, 0.1–5.1 years) for sJIA (3.3 [0.1–5.1] years in the <30 kg group and 3.7 [0.6–5.1] years in the ≥30 kg group). The total follow-up in the safety population was 101.0 patient-years in the <30 kg group and 72.0 patient-years in the ≥30 kg group in pJIA and 57.9 patient-years and 67.6 patient-years, respectively, in sJIA.

All patients experienced at least one AE during the LTE study (Table 2). The most frequent AEs were in the infections and infestations system organ class (Supplementary Table S1, available at *Rheumatology* online); rates in pJIA were 113.9 per 100 patient-years (95% CI: 94.0, 136.7) in the <30 kg SC-TCZ Q3W group and 90.3 per 100 patient-years (95% CI: 69.7, 115.1) in the ≥30 kg SC-TCZ Q2W group, and rates in sJIA were 165.7 per 100 patient-years (95% CI: 134.2, 202.3) in the <30 kg SC-TCZ Q10D/Q2W group and 144.9 per 100 patient-years (95% CI: 117.6, 176.6) in the ≥30 kg SC-TCZ QW group.

**Table 2.** Safety (LTE study)^a^

	pJIA *N *=* *44	sJIA *N *=* *38
Treatment duration (total patient-years of follow-up), median (range), years	4.6 (0.3–5.1)	3.4 (0.1–5.1)
Duration in study, years		
Total patient-years	173.0	125.6
Median (range)	4.6 (0.4–5.2)	3.5 (0.2–5.2)
AEs, rate/100 patient-years (95% CI)		
Phase 1b OL trial	805.1 (728.7, 887.3)	1200.3 (1103.0, 1303.8)
LTE study	417.9 (388.0, 449.5)	448.4 (412.1, 487.0)
SAEs, rate/100 patient-years (95% CI)		
Phase 1b OL trial	7.9 (2.2, 20.3)	19.3 (8.8, 36.6)
LTE study	4.0 (1.6, 8.3)	4.8 (1.8, 10.4)
Infections and infestations, rate/100 patient-years (95% CI)	104.0 (89.4, 120.4)	154.5 (133.5, 177.8)
Patients with ≥1 SAE, *n* (%)	6 (13.6)	5 (13.2)
Total number of SAE events	7	6
Infections and infestations	5 (11.4)	1 (2.6)
Injury, poisoning, and procedural complications	0	3 (7.9)
Eye disorders	1 (2.3)^b^	0
Investigations	0	1 (2.6)
Nervous system disorders	1 (2.3)^b^	0

a All data are shown in the LTE safety population (all patients who received at least one dose of SC-TCZ and had at least one post-dose assessment in the LTE) unless stated otherwise.

b One event of eye pain reported in conjunction with headache (definitive diagnosis could not be made).

AE: adverse event; LTE: long-term extension; OL: open-label; pJIA: polyarticular juvenile idiopathic arthritis; Q10D: every 10 days; QW, weekly, Q2W: every 2 weeks; Q3W: every 3 weeks; SAE: serious adverse event; SC: subcutaneous; sJIA: systemic juvenile idiopathic arthritis; TCZ: tocilizumab.

Seven SAEs were reported among six patients with pJIA and six SAEs were reported among five patients with sJIA (Table 2; Supplementary Table S2, available at *Rheumatology* online). Serious infections were reported in five patients with pJIA (grade 3 furuncle, grade 3 pneumonia, and grade 1 varicella in the <30 kg SC-TCZ Q3W group; grade 2 appendicitis and grade 3 infectious mononucleosis in the ≥30 kg SC-TCZ Q2W group) and one patient with sJIA (grade 2 mycoplasma pneumonia in the <30 kg SC-TCZ Q10D/Q2W group). Only the event of pneumonia in a patient with pJIA was considered related to study treatment by the investigator. Tocilizumab treatment was interrupted for the events of furuncle, infectious mononucleosis, and varicella in pJIA and the event of mycoplasmal pneumonia in sJIA; however, none of these serious infections resulted in study discontinuation. All serious infections were reported as resolved, with the furuncle event resolved with sequelae.

Injection site reactions were reported in nine patients with pJIA (three in the <30 kg SC-TCZ Q3W group, six in the ≥30 kg SC-TCZ Q2W group) and three patients with sJIA (all in the ≥30 kg SC-TCZ QW group); all injection site reactions were non-serious (grade 1 or 2) and none necessitated dose interruption or withdrawal from treatment. Three patients with pJIA and two patients with sJIA experienced potential hypersensitivity reactions; none of these events were serious (all grade 1 or 2) or clinically significant (i.e. did not lead to changes in study treatment or withdrawal) and all resolved without sequelae.

There were no deaths, anaphylactic reactions, serious hepatic events, serious hypersensitivity reactions, serious bleeding events, demyelination events, gastrointestinal perforations, opportunistic infections, malignancies, myocardial infarctions, or strokes reported over the course of the study. There were no cases of macrophage activation syndrome reported during the LTE, and no events consistent with DRESS syndrome were found in *Ad hoc* analysis. Neutropenia AEs were reported in 17 patients overall (nine pJIA [six grade 3, one grade 4] and eight sJIA [three grade 3]); however, no serious infections were reported within 30 days of any neutropenia AE. Thrombocytopenia AEs were reported in two patients with sJIA in the ≥30 kg SC-TCZ QW group (both grade 1).

Of the 13 SAEs reported during the LTE, six occurred during concurrent methotrexate and glucocorticoid treatment (one serious infection), one occurred during methotrexate treatment (serious infection), one occurred during glucocorticoid treatment, one occurred in a patient who received methotrexate before study entry (serious infection) and four occurred without methotrexate or glucocorticoid treatment (all serious infections); five SAEs occurred in patients with body weight <30 kg (three serious infections) and eight SAEs occurred in patients with body weight ≥30 kg (two serious infections) (Supplementary Table S3, available at *Rheumatology* online). Laboratory abnormalities were consistent with those reported in the core studies [7] (Supplementary Data S1, available at *Rheumatology* online).

## Discussion

This long-term analysis of patients with pJIA and sJIA treated with subcutaneous tocilizumab in a clinical trial LTE showed that approved doses were well tolerated. Safety was consistent with the reported safety profile in all SC-TCZ dosing groups in pJIA and sJIA and no new safety signals were observed with long-term SC-TCZ treatment. There were no anaphylactic reactions, and only two patients with pJIA discontinued the LTE for safety reasons. None of the patients with sJIA experienced macrophage activation syndrome.

Previous pharmacokinetic analyses of the phase 1b study data reported that the subcutaneous dosing regimens of 162 mg QW for patients with sJIA weighing ≥30 kg (Q2W for <30 kg) and 162 mg Q2W dosing for patients with pJIA weighing ≥30 kg (Q3W for <30 kg) provided tocilizumab exposures similar to those of approved intravenous tocilizumab regimens [7]. Pharmacokinetic analysis in the LTE showed that tocilizumab exposure was maintained over time within ranges expected to provide clinical benefit in pJIA and sJIA [19]. Pharmacodynamic parameters of interest for tocilizumab (sIL-6R and IL-6, which indicate the mechanism of action, and CRP and ESR) remained stable during the LTE across the pJIA and sJIA body weight dosing groups, suggesting that inflammation continued to be controlled with long-term tocilizumab use. This is consistent with the pharmacodynamic profile of subcutaneous tocilizumab in adults with rheumatoid arthritis [19, 20]. sIL-6R and interleukin-6 levels do not decrease during tocilizumab treatment because the formation of sIL-6R/tocilizumab immune complexes prolongs the elimination half-life of sIL-6R and blocks the consumption of IL-6 by free IL-6R [21]; therefore, they are not suitable biomarkers of the efficacy of tocilizumab. However, IL-6-mediated synthesis of CRP in hepatocytes, a marker of inflammation, is known to be reduced by tocilizumab treatment [22].

The efficacy of SC-TCZ was maintained over the 3-year efficacy assessment period in pJIA and sJIA, as supported by stable JADAS-71. The proportions of patients who achieved inactive disease and clinical remission on treatment were generally stable over time. This indicates that patients with pJIA and sJIA achieved and maintained long-term disease control with SC-TCZ treatment, which may provide important information for future development of treat-to-target approaches [23, 24]. Disease control appeared to be lowest in the pJIA ≥30 kg group; however, considering the small sample size, the higher JADAS-71 at LTE baseline in this group, and the fact that older patients with a higher body weight are more likely to have longer disease duration and received previous disease-modifying antirheumatic drugs, this may be a random effect, and conclusions cannot be drawn.

The results of this study demonstrate that subcutaneous tocilizumab can be administered for a prolonged period of time in the treatment of pJIA and sJIA, confirming it as a long-term treatment option, potentially decreasing the need for hospital visits and reducing absence from school or work for children and their caregivers [9–11]. Limitations of this analysis include open-label administration of study treatment and lack of a control group. The number of patients who remained in the study for 5 years was low (19 patients with pJIA and 6 patients with sJIA); however, most patients who discontinued the study did so to switch to commercially available subcutaneous tocilizumab as stipulated in the protocols. The comparability in exposure and the risk/benefit profile between subcutaneous and intravenous administration of tocilizumab in pJIA and sJIA was previously demonstrated by comparing data from the phase 1b core trials and data from double-blind randomized controlled trials of intravenous tocilizumab in pJIA and sJIA [7].

This LTE study of subcutaneous tocilizumab in pJIA and sJIA adds to the body of evidence for long-term safety and efficacy of biologic therapy, particularly interleukin-6 inhibition, for disease control in JIA [25–28], with no new safety signals identified with long-term subcutaneous tocilizumab treatment.

## Supplementary Material

keae180_Supplementary_Data

## Data Availability

Qualified researchers may request access to individual patient-level data upon publication through the clinical study data request platform (https://vivli.org/). Further details on Roche’s criteria for eligible studies are available here (https://vivli.org/members/ourmembers/). For further details on Roche’s Global Policy on the Sharing of Clinical Information and how to request access to related clinical study documents, see here (https://www.roche.com/research_and_development/who_we_are_how_we_work/clinical_trials/our_commitment_to_data_sharing.htm).
