# Supplementary material for: Long-term efficacy and safety of subcutaneous tocilizumab in clinical trials of polyarticular or systemic juvenile idiopathic arthritis
Source: Rheumatology (Oxford). 2024 Mar 29;63(9):2535–46. doi: 10.1093/rheumatology/keae180 (PMC11371380; doi:10.1093/rheumatology/keae180)
Supplement: keae180_Supplementary_Data [file keae180_supplementary_data.docx]

**Supplementary Materials**

**Supplementary Data S1. Laboratory Assessments**

Most patients had laboratory values within normal ranges at entry to the LTE (baseline). Among the 44 patients with pJIA and 38 patients with sJIA, decrease in neutrophil count after the LTE baseline was reported in 28 patients (63.6%) with pJIA (three grade 1, 16 grade 2, six grade 3, three grade 4) and in 24 patients (63.2%) with sJIA (seven grade 1, eight grade 2, eight grade 3, one grade 4). No serious infections occurred within 30 days of a low neutrophil count. Post-baseline decrease in platelet count was reported in six patients (13.6%) with pJIA (four grade 1, two grade 4) and in 14 patients (36.8%) with sJIA (13 grade 1, one grade 4). No bleeding events occurred within 30 days of a low platelet count. In patients with pJIA, 19 (43.2%) had post-baseline alanine aminotransferase (ALT) elevation (17 grade 1, two grade 2), 10 (22.7%) had post-baseline aspartate aminotransferase (AST) elevation (all grade 1), and seven (15.9%) had post-baseline bilirubin elevation (three grade 1, four grade 2). In patients with sJIA, 13 (34.2%) had post-baseline ALT elevation (10 grade 1, two grade 2, one grade 4), 10 (26.3%) had post-baseline AST elevation (eight grade 1, one grade 2, one grade 3), and five (13.2%) had post-baseline bilirubin elevation (two grade 1, three grade 2). No patients with pJIA or sJIA met the laboratory criteria for Hy’s law (≥3 × upper limit of normal ALT or AST accompanied by ≥2 × upper limit of normal bilirubin) and there were no serious hepatic AEs. Newly occurring post-baseline lipid elevations occurred in eight patients (18.2%) with pJIA (four with total cholesterol ≥200 mg/dL, four with LDL ≥130 mg/dL) and in 11 patients (28.9%) with sJIA (seven with total cholesterol ≥200 mg/dL, four with LDL ≥130 mg/dL).

**Supplementary Table S1.** Most frequent AEs by system organ class (reported in ≥30% of patients) and preferred term (reported in ≥20% of patients)

|  | **pJIA**  ***N* = 44** | **sJIA**  ***N* = 38** |
| --- | --- | --- |
| Patients with ≥1 AE | 44 (100) | 38 (100) |
| Infections and infestations | 42 (95.5) | 33 (86.8) |
| Nasopharyngitis | 19 (43.2) | 15 (39.5) |
| Gastroenteritis | 14 (31.8) | 3 (7.9) |
| Upper respiratory tract infection | 6 (13.6) | 13 (34.2) |
| Musculoskeletal and connective tissue disorders | 29 (65.9) | 19 (50.0) |
| Arthralgia | 16 (36.4) | 10 (26.3) |
| Gastrointestinal disorders | 25 (56.8) | 19 (50.0) |
| Diarrhoea | 13 (29.5) | 7 (18.4) |
| Vomiting | 12 (27.3) | 7 (18.4) |
| Respiratory, thoracic, and mediastinal disorders | 22 (50.0) | 17 (44.7) |
| Cough | 12 (27.3) | 9 (23.7) |
| Oropharyngeal pain | 9 (20.5) | 8 (21.1) |
| General disorders and administration site conditions | 21 (47.7) | 16 (42.1) |
| Pyrexia | 10 (22.7) | 10 (26.3) |
| Injury, poisoning, and procedural complications | 20 (45.5) | 17 (44.7) |
| Skin and subcutaneous tissue disorders | 18 (40.9) | 14 (36.8) |
| Rash | 6 (13.6) | 8 (21.1) |
| Nervous system disorders | 14 (31.8) | 8 (21.1) |
| Headache | 10 (22.7) | 8 (21.1) |

Data are shown as number (%) of patients with the most frequently reported AEs according to system organ class (cutoff for most frequent, ≥30% of patients) and preferred term (cutoff for most frequent, ≥20% of patients) in either JIA category. Multiple occurrences of AEs by system organ class or preferred term in an individual patient were counted only once.

AE: adverse event; pJIA: polyarticular juvenile idiopathic arthritis; sJIA: systemic juvenile idiopathic arthritis.

**Supplementary Table S2.** AEs according to body weight groups (LTE study)^a^

|  | **pJIA (*N* = 44)** | | **sJIA (*N* = 38)** | |
| --- | --- | --- | --- | --- |
|  | **<30 kg SC-TCZ Q3W**  ***n* = 24** | **≥30 kg SC-TCZ Q2W**  ***n* = 20** | **<30 kg SC-TCZ Q10D/Q2W**  ***n* = 19** | **≥30 kg SC-TCZ QW**  ***n* = 19** |
| Treatment duration (total patient-years of follow-up), years, median (range) | 5.0 (0.6–5.1) | 4.1 (0.3–5.1) | 3.3 (0.1–5.1) | 3.7 (0.6–5.1) |
| Duration in study, years  Total patient-years  Median (range) | 101.0  5.0 (0.7–5.2) | 72.0  4.2 (0.4–5.1) | 57.9  3.2 (0.2–5.0) | 67.6  3.8 (0.7–5.2) |
| AEs, rate/100 patient-years (95% CI)  Phase 1b OL trial  LTE study | 680.5 (584.9, 787.1)  405.9 (367.6, 447.2) | 944.2 (824.8, 1076.0)  434.7 (387.8, 485.6) | 1015.3 (889.1, 1154.3)  464.3 (410.4, 523.2) | 1378.7 (1233.5, 1536.3)  434.7 (386.4, 487.4) |
| SAEs, rate/100 patient-years (95% CI)  Phase 1b OL trial  LTE study | 7.5 (0.9, 27.2)  3.0 (0.6, 8.7) | 8.4 (1.0, 30.3)  5.6 (1.5, 14.2) | 30.5 (12.3, 62.8)  6.9 (1.9, 17.7) | 8.4 (1.0, 30.4)  3.0 (0.4, 10.7) |
| Infections and infestations, rate/100 patient-years (95% CI ) | 113.9 (94.0, 136.7) | 90.3 (69.7, 115.1) | 165.7 (134.2, 202.3) | 144.9 (117.6, 176.6) |
| SAEs, *n* (%) | | | | |
| Patients with ≥1 SAE | 3 (12.5) | 3 (15.0) | 3 (15.8) | 2 (10.5) |
| Total number of SAE events | 3 | 4 | 4 | 2 |
| Infections and infestations | 3 (12.5) | 2 (10.0) | 1 (5.3) | 0 |
| Injury, poisoning, and procedural complications | 0 | 0 | 1 (5.3) | 2 (10.5) |
| Eye disorders | 0 | 1 (5.0)^b^ | 0 | 0 |
| Investigations | 0 | 0 | 1 (5.3) | 0 |
| Nervous system disorders | 0 | 1 (5.0)^b^ | 0 | 0 |

^a^All data are shown in the LTE safety population (all patients who received at least one dose of SC TCZ and had at least one post-dose assessment in the LTE) unless stated otherwise.

^b^One event of eye pain reported in conjunction with headache (definitive diagnosis could not be made).

AE: adverse event; LTE: long-term extension; OL: open-label; pJIA: polyarticular juvenile idiopathic arthritis; Q10D: every 10 days; Q2W: every 2 weeks; Q3W: every 3 weeks; QW: weekly; SAE: serious adverse event; SC: subcutaneous; sJIA: systemic juvenile idiopathic arthritis; TCZ: tocilizumab.

**Supplementary Table S3.** SAEs according to methotrexate use, body weight group, and glucocorticoid use

| **SAE** | **Event** | **Weight group at time of the SAE** | **MTX use at time of the SAE** | **GC use at time of the SAE** | **Comments** |
| --- | --- | --- | --- | --- | --- |
| *pJIA:* | | | | |  |
| 1 | Pneumonia  (admitted to hospital overnight with pneumonia) | <30 kg | No | No | Patient was receiving MTX before study entry; however, MTX was not continued after study enrolment |
| 2 | Infectious mononucleosis  (glandular fever) | ≥30 kg | No | No | — |
| 3 | Eye pain  (pain of ocular movement of unknown origin) | ≥30 kg | Yes | Yes | Occurred concurrently with SAE of headache |
| 4 | Headache  (pain of right temple of unknown origin) | ≥30 kg | Yes | Yes | Occurred concurrently with SAE of eye pain |
| 5 | Appendicitis  (acute appendicitis) | ≥30 kg | No | No | — |
| 6 | Furuncle  (furunculosis) | ≥30 kg | Yes | Yes | — |
| 7 | Varicella  (chicken pox) | <30 kg | Yes | No | — |
| *sJIA:* | | | | | |
| 1 | Craniocerebral injury  (traumatic brain injury) | ≥30 kg | No | No | — |
| 2 | Spinal fracture  (multiple spinal fractures) | ≥30 kg | No | Yes | — |
| 3 | Procedural complication  (complication with wisdom teeth intervention) | ≥30 kg | Yes | Yes | — |
| 4 | Pneumonia mycoplasmal  (mycoplasma pneumonia) | <30 kg | No | No | — |
| 5 | Alanine aminotransferase increased (increased ALT) | <30 kg | Yes | Yes | Event occurred concurrently with SAE of increased AST |
| 6 | Aspartate aminotransferase increased (increased AST) | <30 kg | Yes | Yes | Event occurred concurrently with SAE of increased ALT |

ALT: alanine aminotransferase; AST: aspartate aminotransferase; MTX: methotrexate; SAE: serious adverse event.

**Supplementary Table S4.** List of PRINTO and PRCSG Investigators

| **Name** | **Site** |
| --- | --- |
| Rubén Cuttica | Hospital Gral de Niños Pedro Elizalde, Buenos Aires, Argentina |
| Maria Elena Rama | Hospital de Ninos de la Santisima Trinidad, Hematología, Cordoba, Argentina |
| Jonathan Akikusa | Paediatric Rheumatology, Royal Children’s Hospital, Parkville, Victoria, Australia |
| Jeffrey Chaitow | Paediatric Rheumatology, Westmead Hospital, Wahroonga, New South Wales, Australia |
| Claudio Len | Universidade Federal de Sao Paulo – UNIFES, Sao Paolo, Brazil |
| Clovis Artur Silva | Hospital das Clinicas – FMUSP, Sao Paolo, Brazil |
| Heinrike Schmeling | Alberta Children’s Hospital, Calgara, Alberta, Canada |
| Rayfel Schneider | The Hospital for Sick Children, Toronto, Ontario, Canada |
| Isabelle Kone-Paut | CH de Bicêtre; Pediatrie Generale, Le Kremlin Bicêtre, France |
| Markus Hufnagel | Uniklinikum Freiburg Zentrum für Kinderund Jugendmedizin, Pädiatrische Infektio- u. Rheumatologie, Freiburg, Germany |
| Kirsten Minden | Charité Campus, Virchow Klinikum, Berlin, Germany |
| Gerd Horneff | Asklepios Klinik, Zentrum für Allgemeine, Pädiatrie und  Neonatologie, Sankt Augustin, Germany |
| Fabrizio de Benedetti | Irccs Ospedale Pediatrico Bambin Gesu - Dip. Di Medicina, Rome, Italy |
| María del Rocío Maldonado Velázquez | Hospital Infantil De México "Federico Gomez", Rheumatology, Mexico, Mexico |
| Nadina Rubio | Hospital Universitario Dr. Jose Eleuterio Gonzalez, Pediatria, Monterrey, Mexico |
| Ekaterina Alekseeva | SI Sceintific Children Health Center RAMS, Moscow, Russian Federation |
| Agustin Remesal | Hospital De La Paz; Unidad De Reumatologia Pediatrica, Madrid, Spain |
| Alina Boteanu | Hospital Ramon y Cajal, Servicio de Reumatologia, Madrid, Spain |
| Rosa Bou Torrent | Hospital Sant Joan De Deu; Servicio de Reumatologia Pediatrica, Barcelona, Spain |
| Inmaculada Calvo Penades | Hospital Universitario la Fe: Servicio de Reumatologia Pediatrica, Valencia, Spain |
| Athimalaipet V. Ramanan | Rheumatology Department, Bristol Royal Hospital for Children, Bristol, United Kingdom |
| Gavin Cleary | Alder Hey Children's NHS Foundation Trust, Liverpool, United Kingdom |
| Hermine I. Brunner | Pediatric Rheumatology Collaborative Study Group (PRCSG), Cincinnati Children’s Hospital Medical Center, Cincinnati, OH, United States |
| Ginger Janow | Pediatric Rheumatology, Hackensack University Medical Center, Hackensack, NJ, United States |
| Jennifer Weiss | Pediatric Rheumatology, Hackensack University Medical Center, Hackensack, NJ, United States |
| Daniel Lovell | Division of Rheumatology, Cincinnati Children’s Hospital Medical Center, Cincinnati, OH, United States |
| Alan Martin | Healthcare Research Consultants, Tulsa, OK, United States |
| Kabita Nanda | Seattle Children’s Hospital, Seattle WA, United States |
| Linda Wagner-Weiner | University of Chicago Hospital, Chicago, IL, United States |
| Sara Stern | Immunology/Rheumatology/Allergy University of Utah, Salt Lake City, UT, United States |
| Andrew Zeft | Cleveland Clinic Foundation, Cleveland, OH, United States |
| Jason Dare | Arkansas Children's Hospital Research Institute, Little Rock, AR, United States |

**Supplementary Figure S1.** CHAQ-DI in patients with pJIA (A) and sJIA (B) in the LTE (intention-to-treat population).


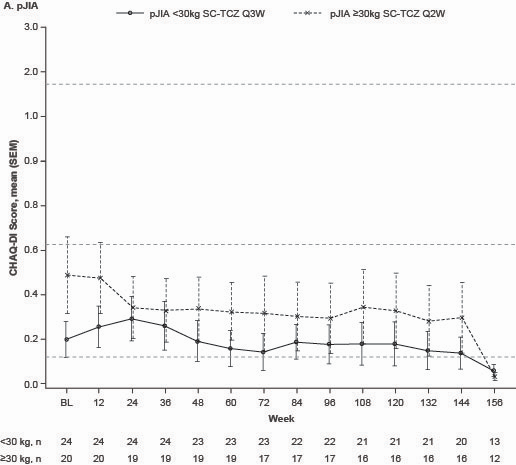


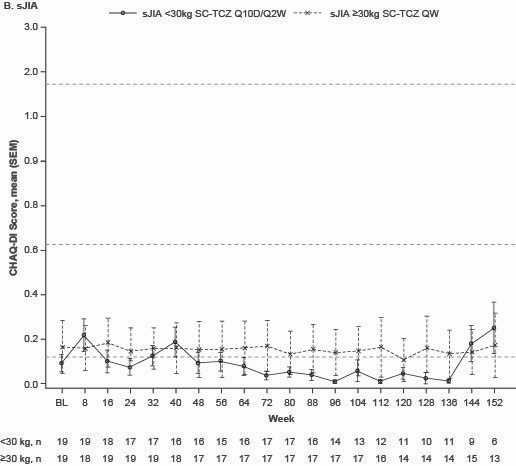


Horizontal dashed lines indicate mild (0.13), mild-to-moderate (0.63), and moderate (1.75) CHAQ disability (Dempster H et al. *Arthritis Rheum* 2001;44:1768–1774).

CHAQ-DI: childhood health assessment questionnaire–disability index; LTE: long-term extension; pJIA: polyarticular juvenile idiopathic arthritis; Q10D: every 10 days; Q2W: every 2 weeks; Q3W: every 3 weeks; QW: weekly; SC: subcutaneous; sJIA: systemic juvenile idiopathic arthritis; TCZ: tocilizumab.

**Supplementary Figure S2.** Patient/parent global assessment VAS in patients with pJIA (A) and sJIA (B) in the LTE (intention-to-treat population).


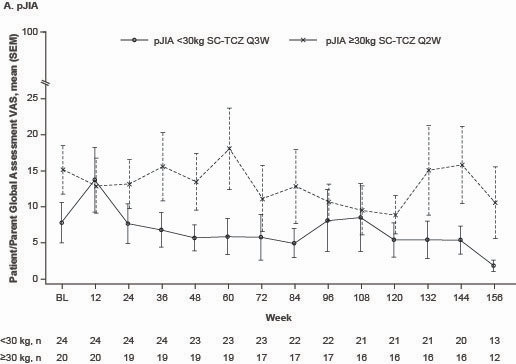


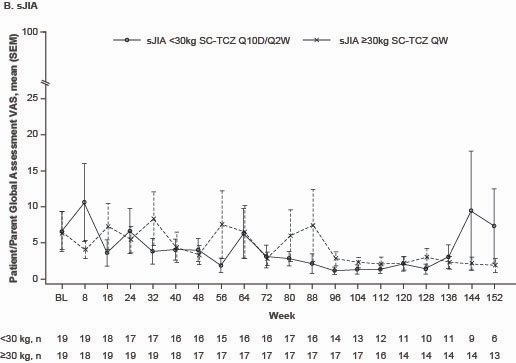


LTE: long-term extension; pJIA: polyarticular juvenile idiopathic arthritis; Q10D: every 10 days; Q2W: every 2 weeks; Q3W: every 3 weeks; QW: weekly; SC: subcutaneous; sJIA: systemic juvenile idiopathic arthritis; TCZ: tocilizumab; VAS: visual analogue scale.
